# The Role of *Porphyromonas gingivalis* Outer Membrane Vesicles in Periodontal Disease and Related Systemic Diseases

**DOI:** 10.3389/fcimb.2020.585917

**Published:** 2021-01-28

**Authors:** Zhiying Zhang, Dongjuan Liu, Sai Liu, Shuwei Zhang, Yaping Pan

**Affiliations:** ^1^ Department of Periodontics, Liaoning Provincial Key Laboratory of Oral Diseases, School and Hospital of Stomatology, China Medical University, Shenyang, China; ^2^ Department of Emergency and Oral Medicine, Liaoning Provincial Key Laboratory of Oral Diseases, School and Hospital of Stomatology, China Medical University, Shenyang, China; ^3^ Department of Dental Materials, Liaoning Provincial Key Laboratory of Oral Diseases, School and Hospital of Stomatology, China Medical University, Shenyang, China

**Keywords:** *Porphyromonas gingivalis*, outer membrane vesicles, virulence factors, periodontal disease, systemic diseases

## Abstract

Periodontal disease is a chronic infectious disease associated with a variety of bacteria, which can cause damage to the periodontal support structure and affect a variety of systemic system diseases such as cancer, cardiovascular disease, diabetes, rheumatoid arthritis, non-alcoholic fatty liver, and Alzheimer’s disease. *Porphyromonas gingivalis* (*P. gingivalis*) is the most important pathogenic bacteria for periodontal disease. It can produce outer membrane vesicles (OMVs) and release them into the environment, playing an important role in its pathogenesis. This article focuses on *P. gingivalis* OMVs, reviews its production and regulation, virulence components, mode of action and related diseases, with a view to providing new ideas for the prevention and treatment of diseases related to *P. gingivalis* infections.

## Introduction

Periodontal disease is a chronic infectious disease associated with a complex of bacterial species leading to the destruction of periodontal structures, including gingiva, periodontal ligament, alveolar bone and cementum (Patini et al., 2018). The initial stage of periodontal disease is gingivitis, which gradually develops into periodontitis as the disease progresses. It can cause gingiva bleeding, tooth mobility and even tooth loss. Apart from oral health issues, many evidences indicate that periodontal disease is tightly bound to systemic diseases, including but not limited to diabetes (Liccardo et al., 2019), cardiovascular disease (Carrizales-Sepulveda et al., 2018), rheumatoid arthritis (Koziel et al., 2014), Alzheimer’s disease (Sochocka et al., 2017), and non-alcoholic fatty liver disease (Alakhali et al., 2018). Specific bacteria form biofilms that accumulate on the tooth surface, interact with host cells, release inflammatory mediators, evade host immune defenses and resist drug action, and play different pathogenic effects in a suitable microenvironment. Therefore, it is very important to study the virulence mechanism of periodontal pathogens for the treatment of periodontal disease and related systemic diseases.

*P. gingivalis* is the most important pathogenic bacteria for chronic periodontitis. It forms the “red complex” with *Tannerella forsythia*, and *Treponema denticola*, which has been the focus of researchers for many years (Darveau et al., 2012). In 1988, Stanley C et al. implanted *P. gingivalis* into the subgingival microbiota of rhesus monkeys and successfully caused periodontitis (Holt et al., 1988). Colonization by *P. gingivalis* leads to impaired innate host defense and promotion of inflammation. These alterations cause quantitative and compositional changes in the subgingival microbiota, which resulting in the emergence of dysbiosis (Hajishengallis et al., 2011; Maekawa et al., 2014). The destruction of inflammatory tissues increases the flow of gingival crevicular fluid (GCF), which brings the degraded collagen and heme-containing compounds into the gingival crevice. These molecules are selectively used by other bacteria, and further develop dysbiotic communities in gingival crevice. In contrast, health-related species are at a disadvantage under this environmental condition, causing imbalances and further exacerbating inflammation, which eventually results in individual periodontitis (Lamont et al., 2018). At the same time, *P. gingivalis* can be detected in other sites such as synovial fluid and plasma, suggesting its potential correlation with systemic diseases (Kriauciunas et al., 2019). It can also interact with the host to promote gene enrichment related to Alzheimer’s disease, diabetes and cardiovascular disease, and even aggravate inflammation at the level of the central nervous system, which is conducive to the occurrence of diseases (Carter et al., 2017; Dioguardi et al., 2020).

With the deepening of research, the OMVs gradually enter people’s field of vision as integral parts of biofilm matrices (Flemming et al., 2007). OMVs are double-layer spherical membrane-like structure with a diameter of about 50 to 250 nanometers that are continuously discharged from the cell surface during the growth of Gram-negative bacteria without loss of membrane integrity (Beveridge, 1999). It is composed of outer membrane proteins, lipopolysaccharides (LPS), phospholipids, DNA, and a part of the periplasm that is enveloped by the outer membrane during the formation process (Cecil et al., 2019). Bacterial OMVs participate in adaption to stress, nutrient acquisition, and communication with host cells and other bacteria (Ellis and Kuehn, 2010). Many enrichment components associated with OMVs are pathogenic factors that contribute to host cell destruction, immune system escape, host cell invasion, or antibiotic resistance. Virulence factors wrapped in OMVs have various advantages including preventing from proteolytic degradation, enhancing long-distance delivery, and coordinating secretion with other bacterial effectors (Bonnington and Kuehn, 2014). In addition, OMVs present a series of natural conformations of surface antigens and have natural characteristics such as immunogenicity, adaptation, and immune cell absorption, making them attractive vaccines against pathogenic bacteria (Pol et al., 2015; Chen et al., 2020). In 1985, Williams and Holt, 1985 first reported that *P. gingivalis* can produce OMVs, but its physiological function and pathogenic mechanism are not clear (Williams and Holt, 1985). In recent years, more and more evidence indicates that *P. gingivalis* OMVs play important roles in the pathogenesis. This review mainly discusses the generation, virulence mechanism and the role of *P. gingivalis* OMVs in periodontal disease and related systemic diseases, and aims to provide new ideas for the prevention and treatment of related diseases.

## Production and Regulation of *Porphyromonas Gingivalis* Outer Membrane Vesicles

Gram-negative bacteria produce OMVs at various stages of growth in various environments, such as infected tissues (Ellis and Kuehn, 2010). OMVs can be formed through different pathways, and they can be produced by different mechanisms even within the same bacterial species (Perez-Cruz et al., 2015). There are various descriptions to explain its generation mechanism. In 1998, Leah Zhou et al. proposed a model for Gram-negative bacteria OMVs formation. During the growth of bacteria, the cell wall is excised and released from the peptidoglycan. If the released muramyl peptides cannot be absorbed, and expansion pressure is always generated on the outer membrane, it will form continuously growing blebs and eventually be shed into the growth medium (Leah Zhou et al., 1998). Therefore, Kuehn and Kesty, 2005 believe that vesicles may be formed at locations where the connection between peptidoglycan and outer membrane is infrequent, absent or broken (Kuehn and Kesty, 2005). In *P. gingivalis*, this mechanism causes the substances located near the outer membrane of the bacteria to be released into the environment in the form of vesicles and act with stronger virulence than the parent bacteria (Mantri et al., 2015).

**Figure 1:**
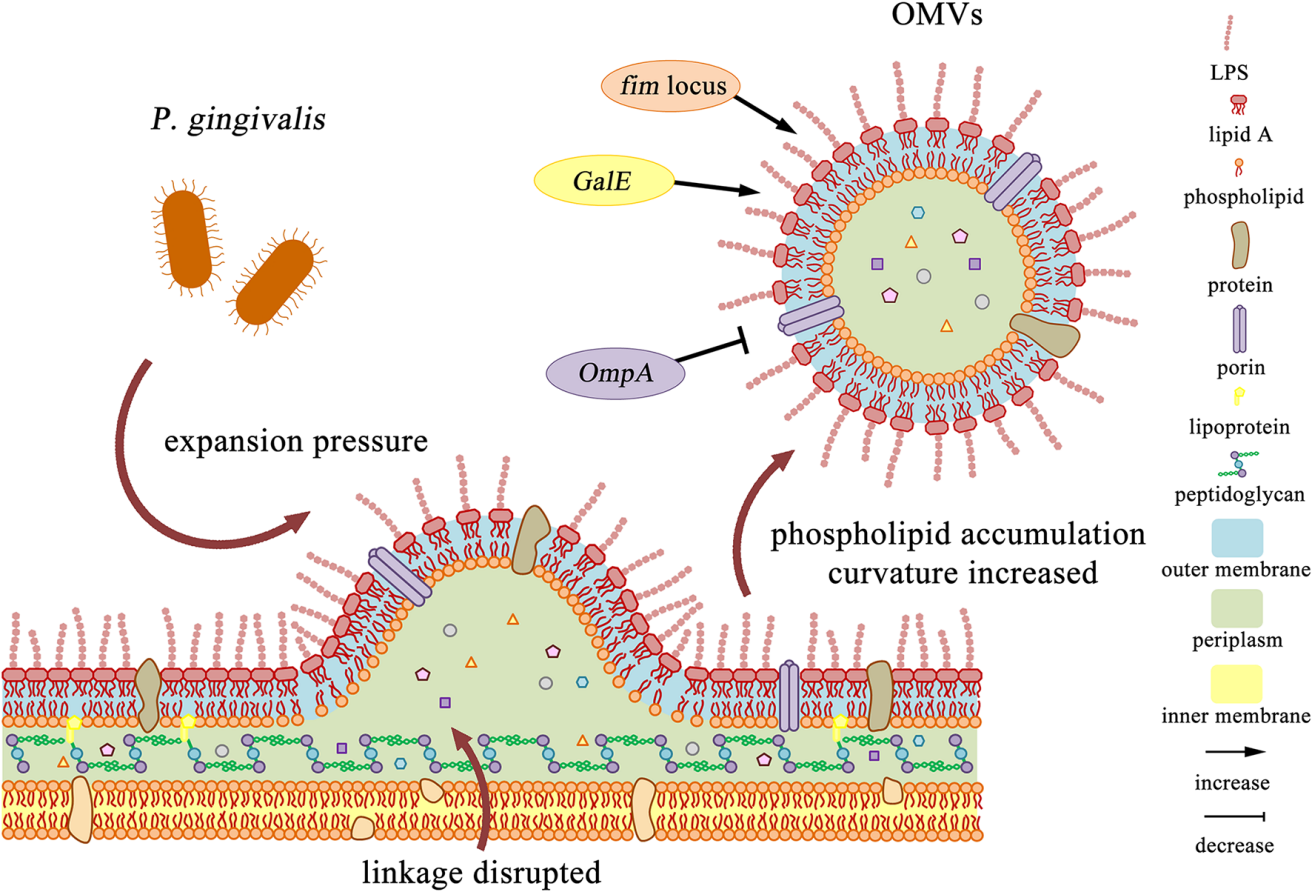
Production and regulation of *P. gingivalis* OMVs. 1. After the cell wall is excised, phospholipids accumulated in the outer membrane leaflets, and the expansion pressure continued to produce, which intensified the further enrichment of outer membrane components. The linkage between the peptidoglycan and the outer membrane layer is disrupted, and finally *P. gingivalis* OMVs are formed. 2. The *fim* locus and *GalE* mutant strains reduced or even eliminated the production of *P. gingivalis* OMVs, while the *OmpA* mutant strain overproduce *P. gingivalis* OMVs.

The production of OMVs can be regulated by microorganisms. In 2016, Roier et al. proposed a general mechanism for OMVs generation that can be regulated by microorganisms. First, decreased or missing expression *VacJ* and/or *Yrb* genes leads to phospholipid accumulation in the outer membrane. This asymmetric expansion triggers the outward expansion of the outer membrane. Second, the positive and negative curvatures on the outer membrane will cause further enrichment of phospholipids and support the budding of the outer membrane, which will eventually be released to form OMVs. Finally, the released OMVs are enriched in phospholipids present in the outer membrane. This biogenesis mechanism of OMVs based on phospholipid accumulation can cooperate with all other OMVs formation models proposed so far (Roier et al., 2016). For *P. gingivalis*, it also has some special regulatory factors. According to the genotype of *FimA*, the main subunit of fimbriae, *P. gingivalis* strains can be divided into six types: I, Ib, II, III, IV, and V. Kerr et al. observed that the surface OMVs of *P. gingivalis* strains ATCC 33277 (type I) and ATCC 49417 (type III) were significantly more than those of W83 strain (type IV) (Kerr et al., 2014). Mantri et al. also found that the OMVs produced by the *FimA* mutant and the *FimR* mutant were much fewer than the parent strain 33277. It indicates that production and pathogenicity of *P. gingivalis* OMVs may mainly depend on the expression of the *fim* locus (Mantri et al., 2015), which may be due to changes in the envelope structure or reduced membrane stability (Baker et al., 2014). Moreover, Nakao et al. found the *GalE* mutant of *P. gingivalis* produced little or no OMVs (Nakao et al., 2011). On the contrary, OMVs were overproduced around the *OmpA* mutant of *P. gingivalis* (Iwami et al., 2007) (**Figure 1**).

Researchers believed that the formation of vesicles is related to the protective mechanism of bacteria. McBroom and Kuehn, 2007 demonstrated that the amount of OMVs released is directly related to the level of protein accumulation in the cell envelope. After being attacked by stressors or accumulating toxic misfolded proteins, the misfolded protein mimics are preferentially packaged into OMVs for removal, which indicates that the process of vesicle formation can selectively eliminate unwanted substances to increase the survival rate of bacteria (McBroom and Kuehn, 2007). On the other hand, Xie suggests that OMVs carry some antigenic substances of their parent bacteria, which can act as bait to interact with the host or drugs, thereby promoting the survival of *P. gingivalis* in oral environment (Xie, 2015).

## Contents and Virulence Factors

*P. gingivalis* OMVs are small and adherent. It is found that the ratio of cells to OMVs is about 1:2,000 (Cecil et al., 2016). They are more stable since they are not affected by host-derived proteases. Compared to the parent *P. gingivalis*, OMVs can better penetrate deep tissues and activate an inflammatory host response (O’Brien-Simpson et al., 2009), and the way they exert their virulence depends largely on lipids, proteins, and nucleic acids (Gui et al., 2016). The components of virulence-related and transport factors contained in OMVs are dynamic and cannot simply reflect changes on the cell surface, and can be enriched according to different growth conditions (Veith et al., 2018).

**Figure 2:**
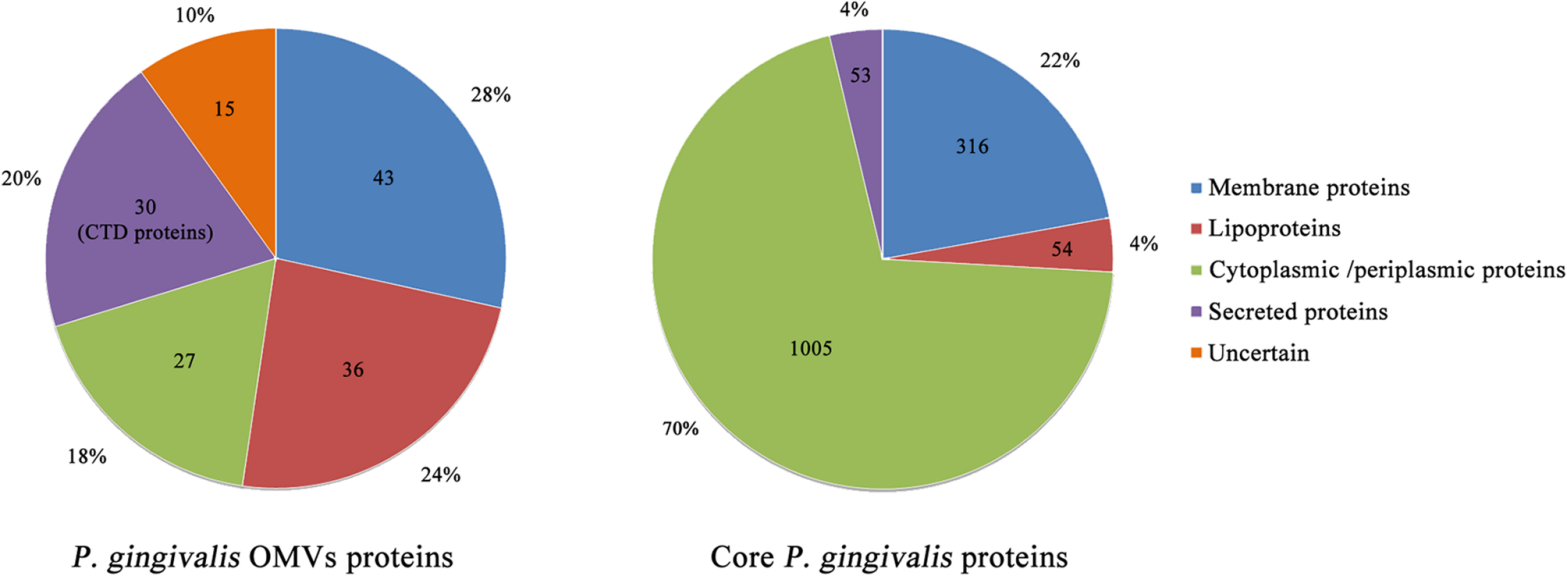
Overview of localization of *P. gingivalis* OMVs and core *P. gingivalis* proteins. The data in the figure show the number of proteins resident at particular locations in the *P. gingivalis* OMVs proteome and the core *P. gingivalis* proteome. Most proteins of the *P. gingivalis* OMVs are membrane proteins, lipoproteins, and extracellular proteins. Among them, all 30 extracellular proteins exhibit CTD secretion signals, including well-studied gingipains, Mfa5, A-LPS, HBP35, CPG70, PPAD, etc. Therefore, *P. gingivalis* OMVs may play a key role in the influence of *P. gingivalis* on the host.

*P. gingivalis* can specifically concentrate considerable virulence factors in the form of OMVs and release them to the environment. Veith et al. performed a proteomics analysis of *P. gingivalis* OMVs and identified a total of 151 proteins, almost all of which were derived from the outer membrane or periplasm, and its protein composition is different from its parent bacteria (Giorgio Gabarrini et al., 2020) (**Figure 2**). Of all the 151 proteins, 30 exhibited CTD secretion signals and localized them on the surface of the vesicles, while 79 and 27 were localized in the vesicle membrane and lumen, respectively, and 15 were of uncertain location (Veith et al., 2014) (**Figure 2**). It is found that all CTD proteins and other virulence factors are abundantly enriched in the OMVs, while proteins that exhibit *OmpA* peptidoglycan binding motif and *TonB*-dependent receptors are preferentially retained on the outer membrane of *P. gingivalis* (Veith et al., 2014). CTD protein is a protein containing a C-terminal domain (CTD), which can be secreted by the type IX secretion system (T9SS). CTD can direct the protein to the outer membrane translocon of *P. gingivalis*. After the secreted protein is modified in the inner membrane and translocated across the outer membrane, the CTD is removed by a protease with sortase-like activity and the secreted protein is modified by A-LPS. Thereafter, the secreted protein can be released into the environment or anchored on the surface of the bacteria (Lasica et al., 2017; Kim and Davey, 2020). CTD proteins include the well-studied gingipains, Mfa5, A-LPS, HBP35, CPG70, PPAD, etc (Shoji et al., 2011). Gingipains are a group of proteases, including Kgp and Rgps, which constitute a major virulence factor of *P. gingivalis* (Sato et al., 2010). They can promote the destruction of supporting bones and tissues in the oral cavity, thereby promoting *P. gingivalis* cell spread throughout periodontal tissue and host cell invasion (Li and Collyer, 2011). Haurat et al. considered that *P. gingivalis* could selectively package certain outer membrane proteins, mainly gingipains, into OMVs and exclude other abundant outer membrane proteins from the OMVs, such as PG0694 and PG0695. However, in the case of *WaaL* mutant, no gingipains were detected in OMVs, which may be related to the specific mechanism of protein sorting during the formation of *P. gingivalis* OMVs (Haurat et al., 2011). Data show that gingipain levels on OMVs are three to five times higher than their parent bacteria (Mantri et al., 2015). In addition to gingipains, heme-binding lipoproteins *HmuY* and *IhtB* are selectively enriched on the surface of *P. gingivalis* OMVs, while their cognate *TonB*-linked transmembrane transport proteins *HmuR* and *IhtA* remained on the surface of *P. gingivalis* (Veith et al., 2014). Heme is an essential growth factor and virulence regulator of *P. gingivalis*. It can be obtained from hemoglobin through the synergistic effect of heme-binding proteins and gingipains (Smalley et al., 2011). Among *P. gingivalis* OMVs, the most up-regulated proteins in response to heme limitation are the proteins involved in the binding and transport of heme, and the 4 most up-regulated proteins in the case of heme excess constitute the putative heme efflux system (Veith et al., 2018). The preferential packaging of these heme-binding proteins and the gingipains on OMVs indicates that OMVs can achieve micronutrient capture by obtaining heme (Gui et al., 2016). *P. gingivalis* OMVs can return the heme-loaded OMVs to the biofilm and provide many other subgingival plaque bacteria with these micronutrients, thereby providing community benefits that allow other species to proliferate. Similarly, the oligo-, monosaccharides, peptides, and amino acids produced by the activity of hydrolases located in OMVs can also be used in other bacteria (Elhenawy et al., 2014). This may be one of the mechanisms by which *P. gingivalis* acts as a key pathogen producing dysbiosis.

In addition to protein components, OMVs also have some noncoding RNAs, which play critical roles in many biological processes, including microRNA(miRNA), long noncoding RNA(lncRNA) and circular RNA(circRNA) (Chew et al., 2018). In 2015, Sjöströmz et al. first reported that RNA is one of the components associated with OMVs (Sjostrom et al., 2015). Recently, a novel class of small RNAs of miRNA size (miRNA-size, called small RNAs or msRNAs) has also been found in several bacteria. Choi et al. listed msRNAs with high clone copy number in *P. gingivalis* OMVs, including P.G_45033, P.G_4378, P.G_122, P.G_16418, and P.G_25037. Subsequent experiments found that these msRNA can be delivered to eukaryotic cells, identify certain potential immune-related target genes, and inhibit the expression of certain cytokines in Jurkat T cells (Choi et al., 2017). High-throughput RNA-seq has revealed that msRNAs may act either as virulence factors or modulator of virulence factors (Diallo and Provost, 2020). However, the specific role of msRNA in *P. gingivalis* OMVs and related mechanisms need to be further explored.

## Roles of *Porphyromonas Gingivalis* Outer Membrane Vesicless in Oral Inflammation Microenvironment

**Figure 3:**
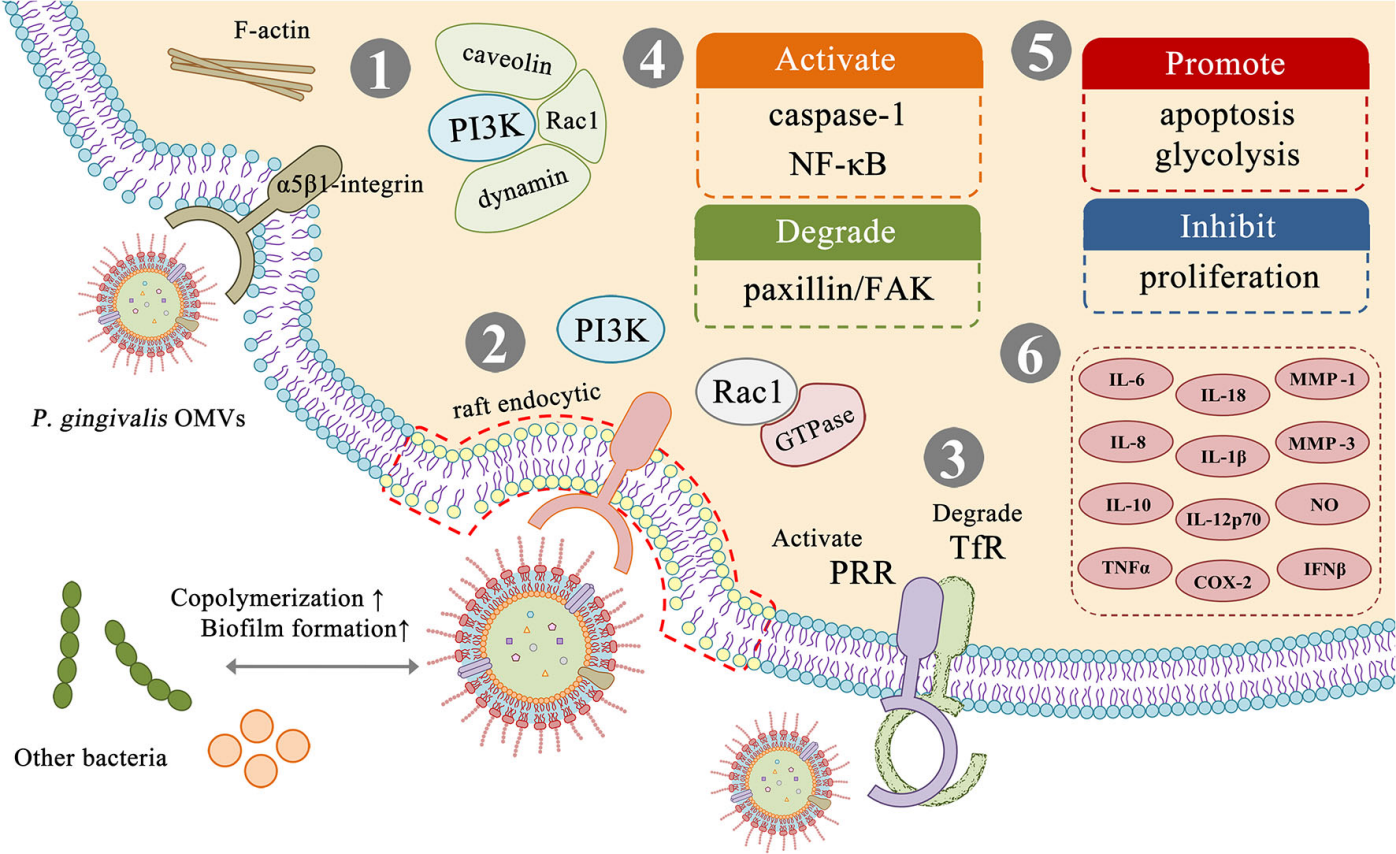
Virulence factors and related effects in *P. gingivalis* OMVs. (1) *P. gingivalis* OMVs can be internalized into cells by an actin-mediated pathway that utilizes host receptors, especially α5β1-integrin, which is controlled by PI3K and depends on caveolin, dynamin, and Rac1. (2) *P. gingivalis* OMVs can be internalized into cells through the fimbria-dependent lipid raft pathway, which is dependent on PI3K and Rac1, and involves various regulatory GTPases. (3) *P. gingivalis* OMVs can exert virulence by affecting different receptors on the host cell surface, such as activating PRR receptors and degrading TfR receptors. (4–6) *P. gingivalis* OMVs can activate or degrade a variety of biologically active substances in host cells, inhibit cell proliferation, promote glycolysis, apoptosis, and cause host cells to produce a variety of inflammatory factors thereby promoting the formation of an inflammatory environment.

Dental plaque formation is an important factor in periodontal disease. Dental plaque is a biofilm formed by the aggregation of various microorganisms, and the interaction between different species is established through specific recognition between adhesins and receptors. Most of the noted adhesins such as *FimA* and *Mfa1* are present in *P. gingivalis* OMVs. As a result, OMVs may represent *P. gingivalis* to communicate with other oral bacteria (Ho et al., 2015) (**Figure 3**). Kamaguchi et al. demonstrated that *P. gingivalis* OMVs strongly promotes coaggregation between *Staphylococcus aureus* with oral microorganisms that do not or only weakly copolymerize with *Staphylococcus aureus*, such as *Streptococcus*, *Actinomyces*, and the mycelium type *Candida albicans* (Kamaguchi et al., 2003). Grenier found that *P. gingivalis* OMVs can mediate the coaggregation between *Treponema denticola* and *Lachnoanaerobaculum saburreum*, and that non-motile bacteria can be transported by carrying spirochetes (Grenier, 2013). *P. gingivalis* OMVs can also inhibit and disperse competitive biofilms in a gingipain-dependent manner, such as biofilms composed of *Streptococcus gordonii*, thereby creating a favorable environment for *P. gingivalis* (Ho et al., 2015). In addition, Inagaki found that *P. gingivalis* OMVs enhance adhesion and invasion of epithelial cells by *Tannerella forsythia* (Inagaki et al., 2006). It can be seen that *P. gingivalis* OMVs can change the composition of plaque biofilm, but its specific mechanism remains to be explored.

Gui concluded that *P. gingivalis* OMVs can be internalized into cells through two different mechanisms (Gui et al., 2016) (**Figure 3**). The first internalization mechanism is considered to be an actin-mediated pathway. *P. gingivalis* OMVs can utilize host cell receptors, especially α5β1-integrin to adhere and trigger the polymerization of F-actin, thus inducing OMVs cellular engulfment. The pathway is controlled by phosphatidylinositol 3 kinase (PI3K), which is dependent on caveolin, dynamin, and Rac1 (Tsuda et al., 2005). The second mechanism is considered to be fimbriae-dependent and mediated through lipid raft endocytosis, it depends on PI3K and Rac1, and involves various regulatory GTPases (Furuta et al., 2009b). *P. gingivalis* OMVs swiftly enter host epithelial cells *via* an endocytosis pathway, survive in the organelles for a period, and are finally sorted to lytic compartments (Furuta et al., 2009b). The choice of endocytosis pathway is based on the different sizes of endocytosis particles (Conner and Schmid, 2003).

*P. gingivalis* OMVs can play a variety of virulence after entering the host cell (**Figure 3**). Nakao et al. found OMVs cause oral epithelial cell detachment in a dose-dependent manner, but this effect can be completely inhibited by arginine-specific gingipain antiserum, suggesting that OMV-associated gingipains were responsible for this activity (Nakao et al., 2014). Bartruff et al. reported that OMVs not only inhibit the proliferation of fibroblast and endothelial cells, but also suppress angiogenesis, resulting in inhibited wound repair in periodontal tissues (Bartruff et al., 2005). These OMVs activate pattern recognition receptors (PRRs) in gingival epithelial cells, leading to cell activation, cytokine secretion or apoptosis (Cecil et al., 2019). Furuta et al. found that OMVs can impair the function of epithelial cells by degrading the signaling molecules required for cell migration such as TfR and paxillin/FAK, leading to cellular impairment (Furuta et al., 2009a). Kou et al. found that after co-culture of immortalized human gingival epithelial cells with *P. gingivalis* OMVs, the inflammation-related factor cyclooxygenase (COX)-2, interleukin (IL) -6, IL-8, matrix metalloproteinase (MMP)-1 and MMP-3 expression levels increased (Kou et al., 2008). Fleetwood et al. found that *P. gingivalis* OMVs can penetrate gingival tissue, causing tissue damage and inflammation. Compared with cells infected with *P. gingivalis*, OMVs stimulated macrophages produce a large amount of TNFα, IL-12p70, IL-6, IL-10, IFNβ, and nitric oxide, and promote the gingival tissue macrophage populations of glycolysis, which leads to apoptosis. They also effectively activated caspase-1, produced numerous IL-1β, IL-18, released LDH, and were positive for 7-AAD, indicating apoptosis (Fleetwood et al., 2017). Cecil et al. found that *P. gingivalis* OMVs can also induce nuclear factor kappa B (NF-κB) activation, thereby exerting immunomodulatory effects on monocytes and macrophages (Cecil et al., 2017). Pro-inflammatory cytokines promote the destruction of connective tissue and alveolar bone resorption, forming the clinical features of chronic periodontitis (Mendes et al., 2015).

Characteristics of *P. gingivalis* OMVs that stimulate the host’s immune response have led researchers to link it to vaccine development. Nakao et al. identified by ELISA that OMVs retained the immunodominant determinant of *P. gingivalis*. Subsequently, they intranasally inoculated OMVs in BALB/c mice, and later detected a significant increase in *P. gingivalis*-specific IgA in the nasal lavage fluid and saliva of mice, as well as serum IgG and IgA (Nakao et al., 2011). Bai et al. suggest that LPS and A-LPS-modified proteins in *P. gingivalis* OMVs carry immune determinants and eventually induce specific antibodies against *P. gingivalis* in mice (Bai et al., 2015). On the other hand, they found that the serum of patients with periodontitis was significantly more reactive to OMV-producing wild type strain than isogenic OMV-depleted strain, indicating that OMVs are highly antigenic (Nakao et al., 2014). Therefore, they believe that OMVs of *P. gingivalis* can be used as a vaccine for the development of periodontal disease.

## *Porphyromonas gingivalis* Outer Membrane Vesicles and Related Systemic Diseases

**Figure 4:**
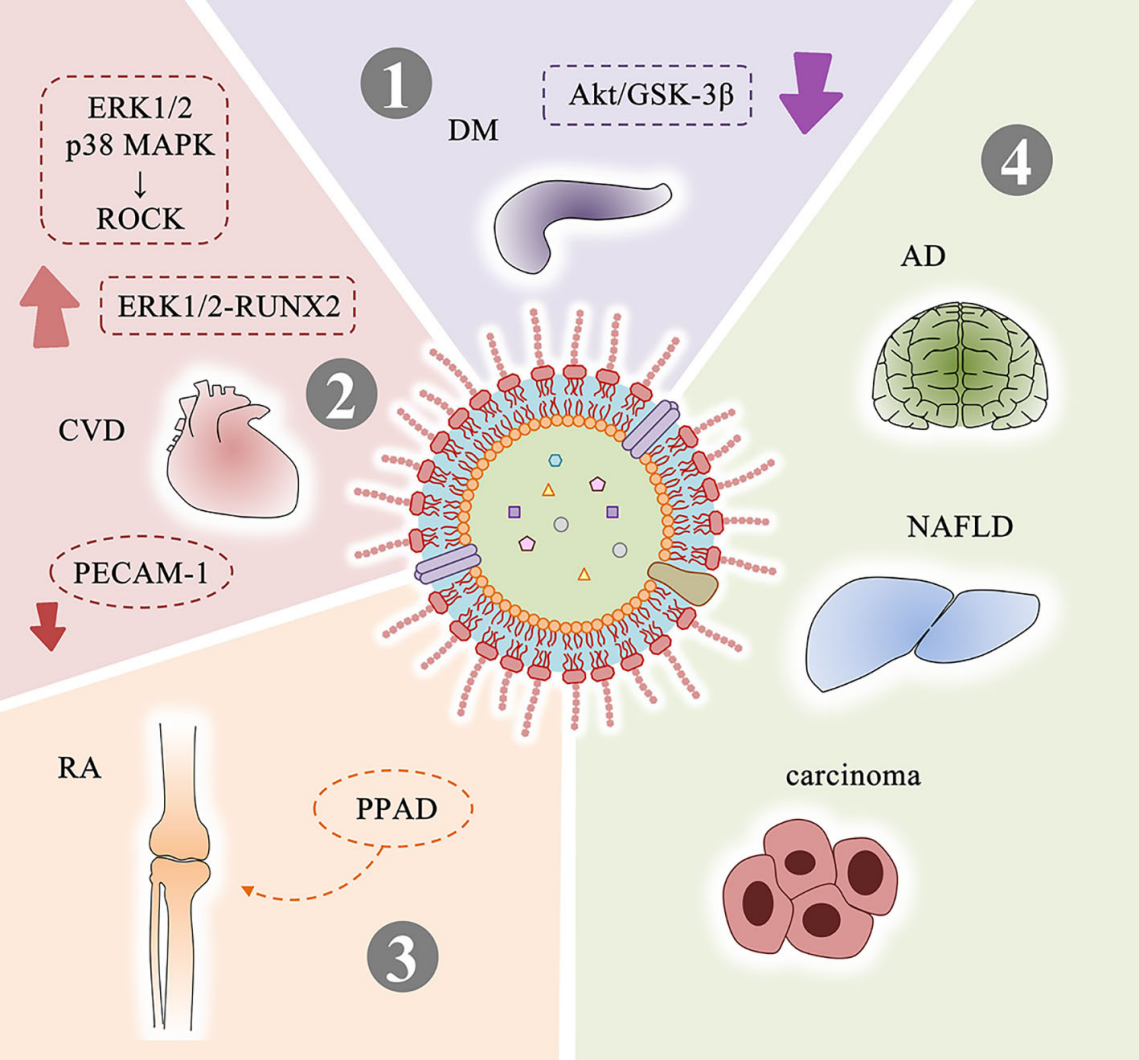
Systemic diseases related to *P. gingivalis* OMVs and possible mechanisms. (1) *P. gingivalis* OMVs attenuate insulin-induced Akt/GSK-3β signaling in hepatic HepG2 cells to promote the development of DM. (2) Mechanisms related to *P. gingivalis* OMVs and CVD: activate ROCK of human umbilical vein endothelial cells through ERK1/2 and p38 MAPK-dependent mechanisms to promote endothelial dysfunction; promote vascular smooth muscle cell calcification through ERK1/2-RUNX2; increase vascular permeability by cleavaging endothelial cell connexins such as PECAM-1. (3) PPAD contained in *P. gingivalis* OMVs is closely related to RA. (4) *P. gingivalis* OMVs may be involved in the progression of AD, NAFLD, and carcinoma. DM, diabetes mellitus; CVD, cardiovascular disease; RA, rheumatoid arthritis; AD, Alzheimer’s disease; NAFLD, non-alcoholic fatty liver disease.

*P. gingivalis* OMVs can migrate to the blood and affect distant tissues and organs (Aguayo et al., 2018). Therefore, OMVs can also play a role in systemic diseases related to *P. gingivalis* infection (**Figure 4**).

### Diabetes Mellitus

Diabetes mellitus (DM) is a group of metabolic diseases characterized by high blood glucose. In the 1960s, researchers discovered a link between DM and periodontal disease (Belting et al., 1964). Multiple studies have demonstrated that this association is bidirectional. On the one hand, people with DM are more likely to suffer from periodontitis (Sergio Guzman et al., 2003). DM stimulates a significant increase in NF-κB expression (Zheng et al., 2018) and activation in periodontal ligament fibroblasts, increases the RANKL/OPG ratios and enhances the expression levels of AGEs, ROS and inflammatory mediators. These factors induce osteoblast apoptosis and osteoclast formation, both increase bone resorption and reduce reparative bone formation, thereby promoting the loss of alveolar bone in periodontitis (Wu et al., 2015). On the other hand, the severity of periodontitis is a factor that affects the development of glycemic control and complications in diabetic patients (Lalla and Papapanou, 2011). Ohtsu et al. found that slight insulin resistance caused by *P. gingivalis* caused an increase in fasting blood glucose levels in streptozotocin-induced diabetic mice (Ohtsu et al., 2019). Seyama et al. confirmed that *P. gingivalis* OMVs can carry active gingipains and delivered to the liver, and attenuated the insulin-induced Akt/glycogen synthase kinase-3β (GSK-3β) signaling in a gingipain-dependent manner in hepatic HepG2 cells. These results indicate that the delivery of gingipains mediated by *P. gingivalis* OMVs causes changes in glucose metabolisms in the liver and promotes the development of DM (Seyama et al., 2020).

### Cardiovascular Disease

Cardiovascular disease (CVD) is still the leading cause of death worldwide according to the World Health Organization (WHO) (Carrizales-Sepulveda et al., 2018). In 1993, DeStefano et al. found for the first time that periodontitis is one of the risk factors for coronary heart disease through a prospective cohort study (Frank DeStefano et al., 1993). Geerts et al. suggested that tooth brushing, chewing, debridement or scaling may cause oral pathogens and their pathogenic factors to enter the bloodstream (Geerts et al., 2002). Results of Zaremba et al. support the possibility that bacteria associated with periodontitis can permeate into coronary vessels as well (Zaremba et al., 2007). Current research shows that the presence of periodontal bacteria in the bloodstream or *in situ* in the vascular lesions is a risk associated with the development of aneurysmal disease (Salhi et al., 2019), and the main feature of *P. gingivalis* infection associated with aneurysms is the proliferation of smooth muscle cells in the distal aorta (Wada and Kamisaki, 2010). It has been found that *P. gingivalis* can be detected in atherosclerotic plaque (Figuero et al., 2011; Szulc et al., 2015), and observed the significance of *P. gingivalis* type II *FimA* for atherosclerosis (Mahalakshmi et al., 2017). Researchers further studied the relationship between *P. gingivalis* OMVs and CVD. Jia et al. found OMVs can activate ROCK of human umbilical vein endothelial cells through ERK1/2 and p38 MAPK-dependent mechanisms, suggesting that they may promote endothelial dysfunction and leading to CVD (Jia et al., 2015). Yang et al. indicated that *P. gingivalis* OMVs promote calcification of vascular smooth muscle cells in a concentration-dependent manner through ERK1/2-RUNX2, which is a hallmark of atherosclerosis (Yang et al., 2016). Farrugia et al. preformed experiments *in vitro* and *in vivo* and confirmed that *P. gingivalis* OMVs significantly increases vascular permeability and enhances vascular edema and mortality in a gingipain-dependent manner. The possible reason involves *P. gingivalis* OMVs cleavage endothelial cell connexins, such as PECAM-1. They believe that the nano-scale size of OMVs will cause proteolytic damage to occur in blood vessels where the parent bacteria cannot access, making OMVs as important as the parent bacteria in the pathogenesis (Farrugia et al., 2020).

### Rheumatoid Arthritis

Rheumatoid arthritis (RA) is a chronic, inflammatory synovitis-based systemic immune disease, which can cause the destruction of articular cartilage and joint capsule, and in severe cases can lead to joint deformities (Leech and Bartold, 2015). Extensive evidence suggests a link between RA and periodontal disease (Kaur et al., 2013). When the periodontal lesions are removed, arthritis remission has been observed in the absence of specific RA therapy (Salemi et al., 2014). *P. gingivalis* DNA exists not only in serum (Menke de Smit et al., 2012) but also in synovial fluid (Reichert et al., 2013). Citrulline is an α-amino acid, patients with RA (about 80%) will develop an immune response against proteins with citrulline. In 1999, McGraw et al. first discovered peptidylarginine deiminase (PPAD) in *P. gingivalis*, which can citrullinate human proteins and potentially contribute to the loss of tolerance to citrullinated proteins in RA (McGraw et al., 1999). The PPAD enzyme produced by *P. gingivalis* not only disturbs the balance of amino acids, but also destroys the entire body’s immune system. It makes the Ag/Ab complexes imbalance, and the human body produces citrullinated antibodies against self-antigens (Kriauciunas et al., 2019). Gabarrini et al. found that PPAD can be associated with OMVs and modified by A-LPS to protect PPAD from proteolytic degradation (Gabarrini et al., 2018). 78 citrullinated proteins were identified in OMVs of *P. gingivalis* W83 wild-type strain, which indicates the association between OMVs and RA (Larsen and Bereta, 2019). 

### Other Systemic Diseases

In addition to the content mentioned above, there are some other systemic diseases that may be associated with *P. gingivalis* OMVs. We have cited Alzheimer’s disease, Non-alcoholic fatty liver disease and carcinoma here.

Alzheimer’s disease (AD) is a neurodegenerative disease characterized by a slow and progressive loss of one or more functions of the nervous system (Du et al., 2018). Carter detected the presence of *P. gingivalis* in AD brains through bioinformatics analysis, indicating that *P. gingivalis* may be associated with AD (Carter, 2017). Dominy et al. succeeded in reducing the bacterial load of the established *P. gingivalis* brain infection by using small-molecule inhibitors against gingipains, blocked Aβ_1–42_ production, reduced neuroinflammation in the hippocampus and rescued neurons (Dominy et al., 2019). This finding demonstrates that *P. gingivalis* and gingipains play a leading role in the pathogenesis of AD, which provides a new conceptual framework for the treatment of AD. As a carrier containing high concentrations of gingipains, *P. gingivalis* OMVs may also play an important role in AD. Studies have confirmed that *P. gingivalis* OMVs-derived LPS can activate glial cells, induce brain inflammation, and correlate with the expression of AD’s marker proteins Aβ and neurofibrillary tangles (Singhrao and Olsen, 2018).

Non-alcoholic fatty liver disease (NAFLD) is a disease in which ≥5% to 10% of liver cells show macroscopic steatosis under an optical microscope without other risk factors for liver disease (Neuschwander-Tetri and Caldwell, 2003). Yoneda et al. found that the detection rate of *P. gingivalis* in patients with NAFLD was significantly higher than that in the control group, and periodontal treatments decreased the serum AST and ALT levels of NAFLD patients (Yoneda et al., 2012). Hisako Furusho et al. first demonstrated in mice that the dental infection of *P. gingivalis* aggravated NASH from simple steatohepatitis to steatohepatitis with fibrosis through a mechanism involving the synergistic interaction between FFA-induced NLRP3 inflammasome activation and the LPS-TLR pathway (Furusho et al., 2013).

Carcinoma is a common cause of death worldwide and can invade and spread to different organs of the body through metastasis (Zhang et al., 2019). There is already a lot of evidence supporting *P. gingivalis* associated with various digestive tract tumors, such as oral squamous cell carcinoma, esophageal cancer, hepatocellular carcinoma, colorectal cancer and pancreatic cancer (Liu et al., 2019). Zhang et al. suggest three mechanisms of oral microbiota in the pathogenesis of cancer, including the induction of chronic inflammatory mediators, the direct impact on the cell cycle and the production of certain carcinogens (Zhang et al., 2018).

There is no report about the relationship between *P. gingivalis* OMVs and NAFLD and carcinoma. It is also very interesting to study the difference between vesicles and parent bacteria on the disease.

## Conclusions

During the growth of *P. gingivalis*, phospholipids accumulate in the outer membrane of *P. gingivalis*, while the outer membrane to swell outwards and eventually pinch off to form OMVs. A large number of pathogenic factors are highly concentrated and protected by the capsule structure to avoid degradation and enhance long-distance delivery, so OMVs can produce stronger virulence than its parent bacteria. These virulence factors mainly include membrane proteins, lipoproteins and extracellular proteins, such as well-studied CTD proteins like gingipains, Mfa5, A-LPS, HBP35, CPG70, PPAD, and heme-binding lipoproteins *HmuY* and *IhtB*. In addition, msRNA is included. It is worth noting that the exact mechanisms of the formation of *P. gingivalis* OMVs are still unknown, so bioinformatics tools have not been or cannot be used to analyze the components in detail, and the existing biochemical analysis is also limited to individual strains. Most of noted adhesins are present in *P. gingivalis* OMVs, which allows *P. gingivalis* OMVs to communicate with other oral bacteria on behalf of their parent bacteria, promote the formation of related bacterial biofilms and inhibit and disperse competing biofilms. On the other side, *P. gingivalis* OMVs can be internalized into host cells through the actin-mediated host receptor pathway and the fimbria-dependent lipid raft endocytosis pathway. It can also activate or degrade a variety of biologically active substances in host cells, inhibit cell proliferation, promote glycolysis, apoptosis, and cause host cells to produce a variety of inflammatory factors thereby promoting the formation of an inflammatory environment. Through long-distance transmission, *P. gingivalis* OMVs can also reach distant target organs and induce related systemic diseases. Many reports indicate the correlation between *P. gingivalis* OMVs and systemic system diseases. Here are a few examples. First, *P. gingivalis* OMVs attenuate insulin-induced Akt/GSK-3β signaling in hepatic HepG2 cells, thereby causing changes in glucose metabolism in the liver and promoting the development of DM. Secondly, *P. gingivalis* OMVs activate ROCK of human umbilical vein endothelial cells through ERK1/2 and p38 MAPK-dependent mechanisms to promote endothelial dysfunction, promote vascular smooth muscle cell calcification through ERK1/2-RUNX2, and increase vascular permeability by cleavaging endothelial cell connexins such as PECAM-1, thereby promoting CVD. Thirdly, *P. gingivalis* OMVs contain PPAD that closely related to RA, and are modified by A-LPS to protect PPAD from proteolytic degradation. However, the relationship between *P. gingivalis* OMVs and other systemic diseases including AD, NAFLD and carcinoma is still little researched, and further exploration is needed. In summary, compared to its parent bacteria, OMVs contain a higher concentration of virulence factors, and a thin membrane is used to protect their smaller structures. These characteristics enable OMVs to achieve long-distance transmission and reach locations where the parent bacteria cannot access, amplifying the pathogenic effects of *P. gingivalis* in the oral environment and even in various parts of the body. This also suggests that the balance of our oral microecological is of great significance to oral and whole body health.

## Author Contributions

ZZ collected the data and drafted the manuscript. DL and SL designed and revised the manuscript. SZ revised the details, and YP guided the completion. All authors approved the final version of the manuscript. All authors contributed to the article and approved the submitted version.

## Funding

This work was supported by the National Natural Science Foundation of China (grants 81870771 and 81800975) and China Postdoctoral Science Foundation (grants 2018M640269 and 2019T120224).

## Conflict of Interest

The authors declare that the research was conducted in the absence of any commercial or financial relationships that could be construed as a potential conflict of interest.
